# Feto-Maternal Trafficking of Exosomes in Murine Pregnancy Models

**DOI:** 10.3389/fphar.2016.00432

**Published:** 2016-11-15

**Authors:** Samantha Sheller-Miller, Jun Lei, George Saade, Carlos Salomon, Irina Burd, Ramkumar Menon

**Affiliations:** ^1^Division of Maternal-Fetal Medicine and Perinatal Research, Department of Obstetrics and Gynecology, University of Texas Medical Branch, GalvestonTX, USA; ^2^Department of Biochemistry and Molecular Biology, University of Texas Medical Branch, GalvestonTX, USA; ^3^Integrated Research Center for Fetal Medicine, Division of Maternal-Fetal Medicine, Department of Gynecology and Obstetrics, Johns Hopkins University, BaltimoreMD, USA; ^4^Exosome Biology Laboratory, Centre for Clinical Diagnostics, University of Queensland Centre for Clinical Research, Royal Brisbane and Women’s Hospital, The University of Queensland, BrisbaneQLD, Australia; ^5^Maternal-Fetal Medicine, Department of Obstetrics and Gynecology, Ochsner Clinic Foundation, New OrleansLA, USA

**Keywords:** parturition, signaling, microvesicles, CD-1 mice, oxidative stress

## Abstract

Timing and initiation of labor are well-orchestrated by signals communicated between the fetal and maternal compartments; however, how these signals are communicated is not completely understood. Fetal exosomes, intercellular signaling vesicles, may play a key role in the process. The objective of this study was to evaluate exosome trafficking *in vivo* from fetal to maternal compartments. Pregnant CD-1 mice were intra-amniotically injected on gestational day 16 and 17 with exosomes isolated from primary human amnion epithelial cells fluorescently labeled with the lipophilic dye 1,1-dioctadecyl-3,3,3,3-tetramethylindotricarbocyanine iodide (DiR). All our analyses were performed on samples collected on Day 18. After 24 h, mice were imaged using Bruker MS FX PRO *In vivo* Imager and tissues were collected. *In vivo* imaging of mouse showed fluorescence in the uterus, on the exosome-injected side whereas the uterine tissues from the uninjected side and saline and dye alone injected animals remained negative. Histological analysis of placenta showed exosome migration from the fetal to the maternal side of the placenta. Fluorescence released from exosomes was seen in maternal blood samples as well as in maternal uterus and kidneys. This study demonstrates that exosomal cargo can be carried through systemic route from the fetal to the maternal side of the uterine tissues during pregnancy, supporting the idea that fetal signals can be delivered via exosomes.

## Introduction

Human parturition is widely accepted as an inflammatory process initiated by environmental, endocrine, and physiological factors, although the precise mechanisms involved are still unclear (Gonzalez et al., 2011; Reinl and England, 2015). Normal term parturition consists of well-orchestrated events involving both fetal and maternal compartments (Blackburn, 2014; Gao et al., 2015). On the maternal side, activation of the decidua, myometrial functional progesterone withdrawal, and cervical ripening are all considered mechanistic signals associated with parturition (Shynlova et al., 2013; Romero et al., 2014). Both endocrine and paracrine fetal biochemical signals released from matured organs, such as increased cortisol production by fetal adrenals or surfactant protein-A from fetal lungs, can induce parturition (Mendelson, 2009; Gao et al., 2015; Reinl and England, 2015; Menon et al., 2016b). Our laboratory has investigated a new fetal signaling mechanism initiated by fetal membrane senescence in response to inflammation and oxidative stress that builds up in the amniotic cavity at term (Menon, 2014; Menon et al., 2014, 2016b; Behnia et al., 2015). This leads to senescence-associated sterile inflammation through the release of inflammatory cytokines, chemokines, matrix degrading enzymes and growth factors, termed senescence-associated secretory phenotype (SASP; Menon et al., 2013, 2016a; Menon, 2014; Behnia et al., 2015). Senescent cells also secrete damage associated molecular patterns (DAMPs), which are well known inflammatory mediators released from dying cells communicating cellular damage (Srikrishna and Freeze, 2009; Garg et al., 2014).

Although the senescent signal action is predominantly localized, these signals of cellular stress may get carried to maternal tissues, signaling fetal maturity and prompting delivery of the fetus (Natasha et al., 2014; Zhang et al., 2014). Distant senescent signaling is likely facilitated through intercellular signaling vesicles called exosomes (Sheller et al., 2016). Exosomes are 30–100 nm endosome-derived vesicles with specific characteristics that separate them from other larger particles such as microvesicles and apoptotic bodies (György et al., 2011; Akers et al., 2013). First described as modulators of the immune response to cancer cells, exosomes have also been found to contribute to angiogenesis and metastasis (Bobrie et al., 2011; Rodríguez et al., 2014). The current research involving exosome signaling in tumorigenesis via immune cell modulation has increased interest in their role in inflammatory disorders, such as asthma, arthritis and inflammatory bowel disease (Eldh et al., 2010; Corrado et al., 2013; Rodríguez et al., 2014). Since inflammation is an underlying theme in the initiation and progression of labor (Shynlova et al., 2013; Gomez-Lopez et al., 2014; Menon, 2014; Srikhajon et al., 2014; Reinl and England, 2015), it is likely that exosomes play an important role in cell signaling during term labor.

Exosome size facilitates transport between cells and tissues, while their contents, which reflect the functional state of the cell of their origin, may regulate the phenotype of the target cell (Raimondo et al., 2011; Kobayashi et al., 2014; Mulcahy et al., 2014). Ongoing studies in our laboratory have shown that myometrial cells treated with exosomes from amnion epithelial cells (AECs) cultured under oxidative stress conditions induce a contractile phenotype through the activation of NFκB and gene transcription activation of contraction associated proteins COX-2 and Connexin 43.

Although studies show exosomes can induce functional changes in myometrial cells, we do not know if the fetal membrane-derived exosomes can reach the maternal tissues to induce labor. The objective of this study was to determine the biodistribution of exosomes *in vivo* in pregnant animal models. By injecting fluorescently labeled amnion cell-derived exosomes into the amniotic fluid of pregnant CD-1 mice, we observed the migration of exosomes from the fetal to the maternal tissues.

## Materials and Methods

### Patient Inclusion Criteria

No subjects were recruited or consented for this study since we used discarded placenta from normal term, not-in-labor cesarean sections that were de-identified before they were received by lab staff, as described previously (Sheller et al., 2016). Placental samples obtained for this study were from the John Sealy Hospital at The University of Texas Medical Branch (UTMB) at Galveston, TX, USA. The collection of placenta was approved by the institutional review board at The University of Texas Medical Branch at Galveston in compliance with all applicable Federal regulations governing the protection of human subjects (#11-251 April 2013). This protocol allowed us to collect discarded placental specimens after normal term cesarean deliveries or vaginal deliveries as an exempt protocol that does not require subject’s consent.

### Isolation and Culture of Human Amnion Epithelial Cells (AECs)

All reagents and media were warmed to 37°C prior to use. The amniotic membrane was processed within 15 min after delivery as described previously (Lim et al., 2013; Menon et al., 2013; Sheller et al., 2016). Primary AECs (*n* = 4) were cultured in T75 flasks containing complete media consisting of Dulbecco’s Modified Eagle Medium: Nutrient Mixture F-12 media (DMEM/F12; Mediatech Inc., Manassas, VA, USA) supplemented with 10% fetal bovine serum (FBS; Sigma-Aldrich, St. Louis, MO, USA), 10% Penicillin/Streptomycin (Mediatech Inc.) and 100 μg/mL epidermal growth factor (EGF; Sigma-Aldrich) at 37°C, 5% CO2, and 95% air humidity to 60–65% confluence.

### Exosome Isolation

Culture media was removed and cells were serum starved for 1 h in DMEM/F12 with 5% pen/strep prior to treatment with exosome-depleted media (DMEM/F12, 5% pen/strep and 10% exosome-depleted FBS) for 48 h. FBS (Sigma-Aldrich) was depleted of exosomes by ultracentrifugation at 100,000 × *g* for 18 h then filter-sterilized with 0.22 μm filter (Millipore, MA, USA) (Soo et al., 2012; Kobayashi et al., 2014). Culture media were collected and stored at -80°C until exosome isolation. Media was thawed overnight then isolated using differential ultracentrifugation as described previously, (Sheller et al., 2016) with the following modifications. After the 2 h 100,000 × *g* centrifugation, the supernatant was removed and the exosome pellet was resuspended in PBS. The sample was then split: half was centrifuged for 1 h at 100,000 × *g* while the other half was labeled with DiR. The final pellets were resuspended in cold PBS and stored at -80°C.

### Labeling of Exosomes with DiR

To fluorescently label exosomes for *in vivo* imaging, we resuspended the pellet centrifuged at 100,000 × *g* for 2 h in 7.0 mL 7.5 μM DiR (Life Technologies, Carlsbad, CA, USA) in PBS. After mixing, the exosomes were incubated in the DiR/PBS solution for 15 min at room temperature in the dark and then ultracentrifuged at 100,000 × g for 1 h. The final pellet was resuspended in 50 μL PBS and stored at -80°C.

### Exosome Characterization Using Transmission Electron Microscopy (TEM) and Western Blot

To show that exosomes isolated from primary AECs exhibit classic exosome shape and morphology, Transmission Electron Microscopy (TEM) studies were performed as described previously (Sheller et al., 2016), with the following modification: exosomes were fixed in 5% buffered formalin; then, 5 μL of exosome suspension were dropped onto the grid and left to dry at room temperature for 10 min. To show exosome and amnion cell markers, we performed a Western blot as described previously (Sheller et al., 2016).

### Animals

All animal procedures were approved by the Animal Care and Use Committee of Johns Hopkins University. Timed-pregnant CD-1 mice, outbred mice reflecting diverse genetic backgrounds in humans, were purchased from Charles River Laboratories (Houston, TX, USA) and received on gestational day 9 (E9). Animals had access to food and water *ad libitum* freely during housing and the experimental period. To determine the biodistribution of exosomes *in vivo*, we anesthetized pregnant CD-1 mice on E16 (*n* = 9) and E17 (*n* = 9) with continuous isoflurane in oxygen and performed intrauterine injections of DiR-labeled exosomes or phosphate-buffered saline solution (PBS).

**FIGURE 1:**
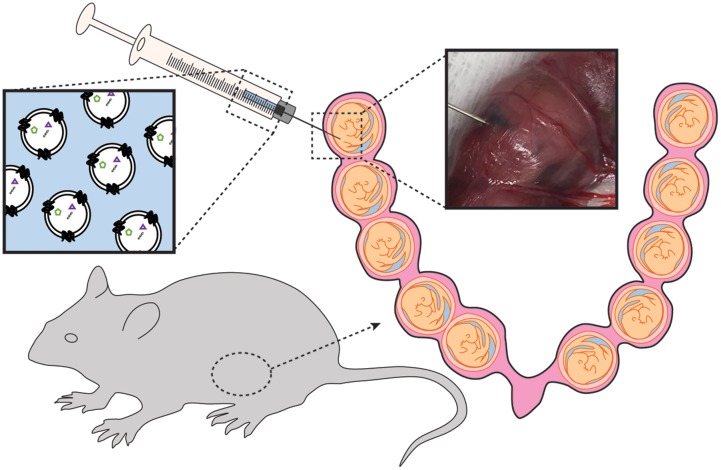
**Illustration of exosomes injected into the amniotic cavity of pregnant mice**.

Mice were subjected to mini-laparotomy, as illustrated in **Figure 1**. Using a Hamilton syringe, saline (*n* = 3 per gestational day) or DiR-labeled exosomes in PBS (*n* = 6 per gestational day) were injected intra-amniotically into each gestational sac on the right side of the cervix (maximum of five injections). The left side of the uterus was not injected and served as an internal control. Surgical incisions were closed, and the dams recovered in individual cages.

After 24 h, animals were imaged under anesthesia on both the dorsal (after hair removal) and abdominal sides using the Bruker In Vivo MS FX PRO Imager (Bruker, Billerica, MA, USA). Upon completion of live imaging, the animals were sacrificed by carbon dioxide inhalation according to the IACUC and American Veterinary Medical Association guidelines. The fetus contained within the uterus was collected in 4% paraformaldehyde (Sigma-Aldrich) and analyzed by histology for the presence of exosomes. Uteruses were also removed from saline- and exosome-injected mice and imaged using IVIS 200 (PerkinElmer, Inc., Waltham, MA, USA). Any image modifications (brightness, contrast, and smoothing) were applied to the entire image using Image J (open source). Maternal plasma was collected for exosome isolation.

Embryos were removed and fixed in 4% PFA at 4°C overnight. The next day, specimens were washed with PBS extensively and immersed in 30% sucrose until saturation, followed by cryosection at a 20-μm thickness. All photographs were taken with Zeiss AxioPlan 2 Microscope System (Jena, Germany). Routine hematoxylin and eosin (H&E) histochemical staining were performed on the neighbor sections.

### Maternal Plasma Exosome Isolation to Localize Trafficking of Exosomes

To determine whether exosomes injected into the amniotic cavity can reach the maternal side via the systemic route, we analyzed serum samples for fluorescently labeled exosomes in maternal serum. Due to the low volume of serum collected, samples were pooled prior to isolation. Exosomes were isolated from maternal plasma, as described above, and the final pellet was resuspended in 100 μL 1:1 glycerol/ethanol solution. To determine if the isolated exosomes contained fluorescence, we pipetted 50 μL of each sample and control in duplicate into a black 96-well plate (Corning) and imaged them on the Biotek Synergy H4 Hybrid (Biotek, Winooski, VT, USA). Wavelength was set for excitation at 745 nm and emission at 779 nm. A serial dilution of DiR in 1:1 glycerol/ethanol solution was used as the positive control, while the glycerol/ethanol solution was used as a negative control. Values were determined using relative fluorescence units (RFU). Upper and lower limits were established by DiR serial dilution (positive control, RFU > 50) and the glycerol/ethanol solution (negative control, RFU < 50). Statistical analysis was not performed as we analyzed pooled sample sets (*n* = 2 in each group) and therefore we report a fold change between the two groups.

## Results

### Amnion Epithelial Cell-Derived Exosome Characterization

**FIGURE 2:**
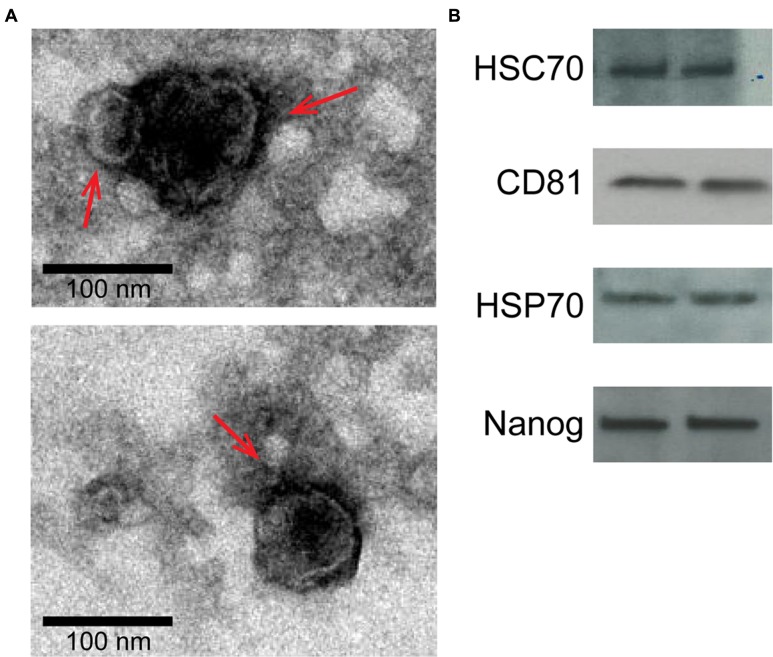
**Characterization of two representative exosome samples isolated from primary amnion cells.** **(A)** Electron microscopy showing cup-shaped vesicles that have a size distribution of 30–100 nm (arrow indicates exosomes; scale bar represents 100 nm). **(B)** Western blot analysis showing the presence of exosome markers HSC70, CD81 and HSP70, as well as embryonic stem cell marker, Nanog, indicating amnion epithelial cell origin.

Isolated exosomes were characterized using TEM and Western blot for exosome and amnion markers as described previously (Sheller et al., 2016). TEM analysis revealed vesicles with classic exosome size and morphology (**Figure 2A**), consistent with previously published reports for exosomes (Redman and Sargent, 2008; Kshirsagar et al., 2012; Salomon et al., 2014; Mitchell et al., 2015). Western blot analysis was performed to determine exosome-enriched markers HSC70, CD81, and HSP70, as well as embryonic stem cell marker Nanog (**Figure 2B**).

### Exosome Trafficking in Pregnant Mice

To determine the trafficking of exosomes injected into the amniotic cavity, we imaged animals 24 h after injection. As a negative control, saline was injected into the amniotic cavity and imaged after 24 h. Fluorescent signals could not be seen in saline-injected mice when imaged (data not shown).

**FIGURE 3:**
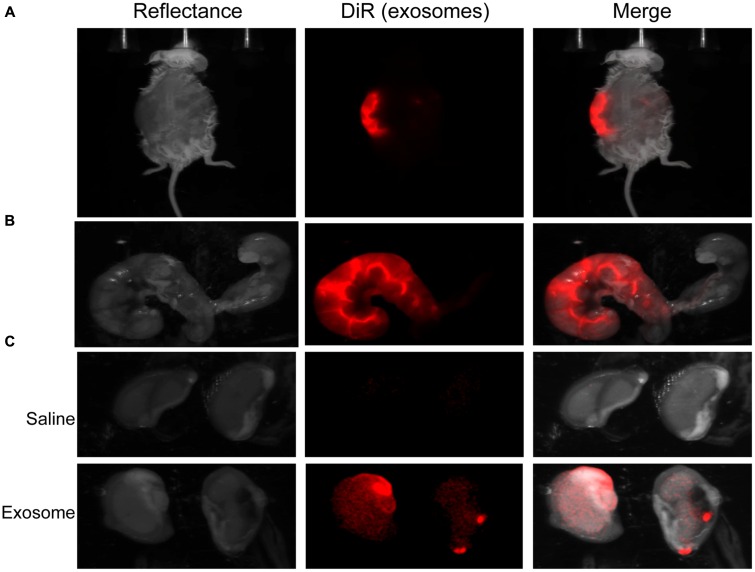
***In vivo* imaging of pregnant mouse 24 h post injection.** Exosomes stained with DiR (red) were injected into five different embryonic sacs on one side of the uterus. **(A)** Dorsal image after removal of hair using Nair. **(B)** Uterus was removed post sacrifice. Red fluorescence indicated embryonic localization of DiR labeled exosomes. Uninjected side (right) lacks fluorescence. **(C)** Kidneys from saline-injected mice (top) do not have fluorescent signal while kidneys from exosome injected mice (bottom) have fluorescent signal. Merge is an overlay of reflectance and DiR.

Fluorescently labeled exosomes were injected into mice and imaged after 24 h. Regardless of the gestational day, exosome-injected mouse images showed fluorescence on the dorsal side (**Figure 3A**), although the signal remained on the injected side. When uteruses were removed and imaged (**Figure 3B**), fluorescent signals were seen only on the injected sides and not on the uninjected sides, confirming the dye stays contained within the exosomes and does not leak from the membrane of the exosomes. On gestational day 18, images of the maternal kidneys from saline- and exosome-injected mice were also taken (**Figure 3C**). Fluorescent signals were not seen in the saline-injected kidneys, but fluorescence was seen in the kidneys of the exosome-injected mice.

**FIGURE 4:**
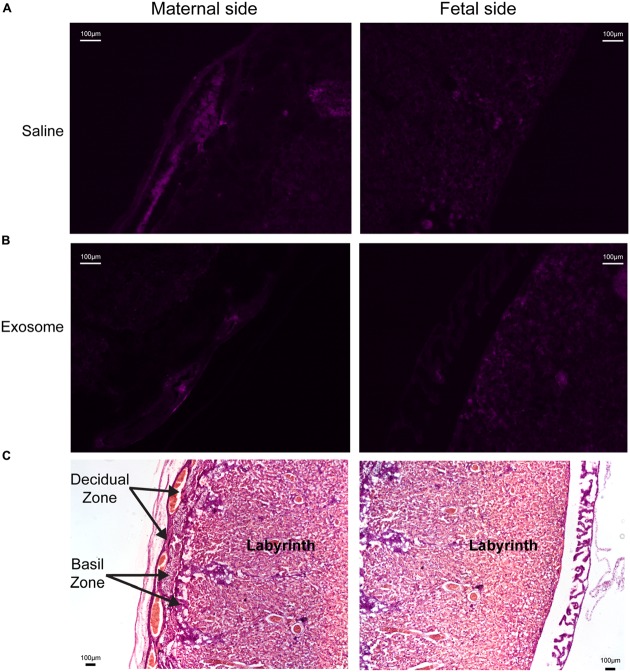
**Maternal and fetal sides of the placenta collected from injected mice on gestational day 17.** **(A,B)** Lack of fluorescence in saline and exosome injected mouse placenta on either maternal or fetal sides. **(C)** H&E staining of maternal and fetal sides of the placenta (scale bars represent 100 μm). Arrows indicate regions of the placenta.

**FIGURE 5:**
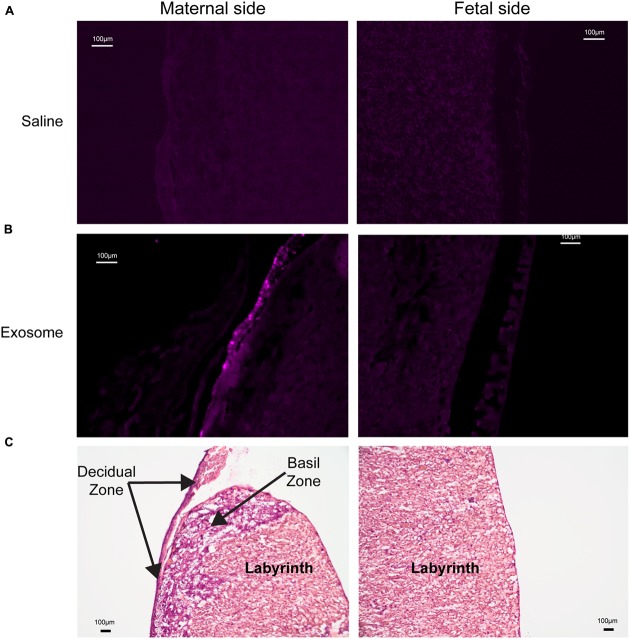
**Maternal and fetal sides of the placenta collected from injected mice on gestational day 18.** **(A)** Saline injected mouse placenta showed no fluorescence on maternal or fetal sides. **(B)** Exosome injected mouse placenta showed fluorescence on maternal side but lacks signal on the fetal side of the placenta. **(C)** H&E staining of maternal and fetal sides of the placenta (scale bars represent 100 μm).

**FIGURE 6:**
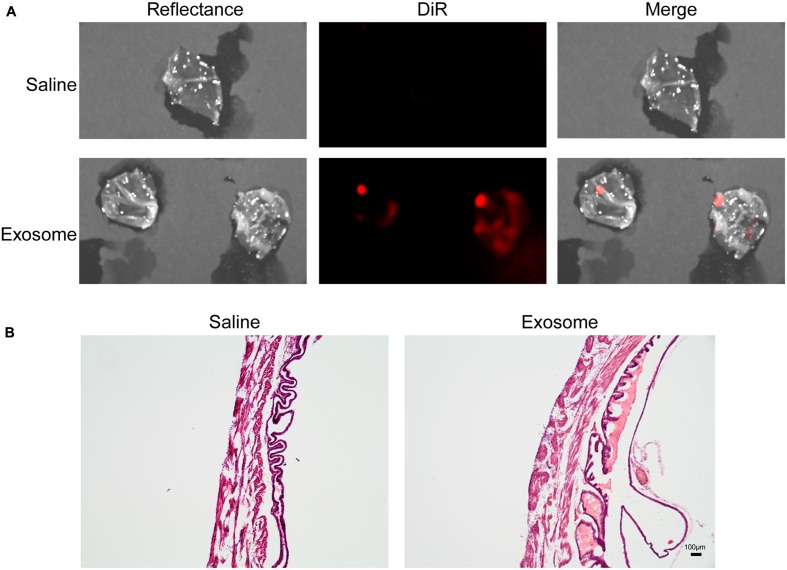
***In vivo* imaging of uterine tissues collected from gestational day 18.** **(A)** Saline-injected (top) mouse uterus showed no fluorescent signal, while exosome-injected (bottom) mouse uterus showed fluorescence, indicating exosomes traffic to the maternal tissues. **(B)** H&E staining of uterus tissue from saline (left) and exosome (right) injected mice (scale bars represent 100 m). Merge is an overlay of reflectance and DiR.

Histologic analyses of placental and uterine tissues were performed to observe exosome trafficking in the reproductive tissues. On E17, the collected exosome- and saline-injected mouse placentas showed no signals (**Figure 4**); however, on E18, signals emerging from exosomes can be seen on the maternal side of the placentas, whereas signals were not seen on the fetal side or in the saline-injected placentas (**Figure 5**). *In vivo* imaging of the uterine tissue showed saline-injected uterine tissue did not have fluorescent signals, whereas uterine tissues from the exosome-injected animals showed fluorescent signals (**Figure 6**). Fluorescent signals were validated using the Biotek Synergy H4 Hybrid, in which the exosome-injected uterine tissues on day 18 had sixfold higher RFU than the saline-injected tissues (data not shown).

### Exosomes Traffic to the Maternal Serum

To validate the observations above and trafficking of exosomes through a systemic route, we analyzed exosomes isolated from maternal serum for fluorescence. Exosome injection solution was also analyzed for fluorescence to ensure exosomes were successfully labeled. Serum from saline-injected mice on E17 and E18, as well as from mice injected with exosomes on E17, had an RFU below 50, similar to the negative control (glycerol/ethanol solution). The serum from the exosome-injected mice on day 18 had an RFU above 50, indicating the exosomes from the amniotic fluid can traffic through the maternal serum later in pregnancy and may be dependent on timing. We report a 2.3-fold higher RFU in the serum of animals injected with DiR labeled exosomes compared to saline controls.

In summary, we demonstrated that exosomes may diffuse to the placental side where they can reach maternal tissues through a systemic route.

## Discussion

It is generally considered that the timing and initiation of labor are well orchestrated by communications between the fetus and the mother (Smith et al., 2002; Challis et al., 2005). However, how these signals are communicated between the fetal and maternal compartments is poorly understood. As intercellular signaling vesicles that can travel long distances through tissues and fluids (Raposo and Stoorvogel, 2013; Mincheva-Nilsson and Baranov, 2014; Bátiz et al., 2015; De Toro et al., 2015), exosomes may be carriers of these signals.

This study was performed to observe exosome trafficking *in vivo*. After labeling exosomes with the near-infrared dye DiR, we determined that exosomes injected intra-amniotically into pregnant mice can be imaged and monitored for their migration. DiR-labeled exosomes injected on E17 were observed in the maternal plasma and kidneys and on the maternal side of the placenta and uterus on E18, indicating migration from the amniotic cavity to the maternal side. Our study shows that exosomes, which potentially carry signals for the initiation of parturition, can traffic from the amniotic fluid into the placenta and systemically spread through circulation.

Exosomes are characterized by their contents, which reflect the physiological status of the origin cell and can regulate the phenotype of the target cell (Kambe et al., 2014; Bátiz et al., 2015; Sheller et al., 2016; Willms et al., 2016). At term, oxidative stress and inflammation build up in the amniotic cavity, causing cellular senescence of the fetal membranes and subsequent release of signals of cellular damage (Menon, 2014; Behnia et al., 2015, 2016; Polettini et al., 2015; Menon et al., 2016b). Senescent signal action is primarily localized, although we have shown signals of cellular damage are also packaged into exosomes from AECs treated with the oxidative stress inducer cigarette smoke extract (Sheller et al., 2016). It is likely that exosomes carrying signals of cellular damage can reach maternal tissues and contribute to parturition.

Though our study answers basic questions about exosome trafficking *in vivo*, it does not include activation of inflammatory pathways involved in the initiation and progression of labor. We only allowed for 24 h prior to imaging and tissue collection, which may not be sufficient time for exosomes to migrate to the target tissues or cause functional changes. Determination of initiation of parturition at term or preterm based on signals carried by exosomes was beyond the scope of this study. Exosomes were stored at -80°C until the day of injection. Although the stability of amnion epithelial cell-derived exosomes during freeze-thaw cycles has been not been evaluated for this study, there have been published reports demonstrating exosome stability in various biological fluids from pregnancy (Sarker et al., 2014). The number of exosomes injected was random, which may have an effect on the trafficking and eventual response to exosome signaling. Our ongoing studies using a specific number of exosomes will determine the effect of the quantity required to cause a functional change and specific pregnancy outcomes like preterm or term parturition. Future studies using live imaging will also determine the timing required for exosome migration between feto-maternal compartments. We will also understand differences between exosomes from amnion cells grown under standard conditions and oxidative stress conditions, including activation of inflammatory pathways related to parturition and preterm birth rates.

In summary, we have demonstrated that exosomes injected into the amniotic cavity of pregnant mice can traffic to the maternal tissues. Specifically, exosomes injected on gestational day 17 migrated to the maternal side of the placenta, the maternal serum, and the maternal kidneys. So we propose that exosomes can traverse through various tissue layers and or it can reach various tissues through a systemic route. Although not tested, the diffusion of exosomes through various layers could be a concentration dependent process and the quantity very well may determine the fetal signal strength. Oxidative stress is expected to increase exosome numbers (Salomon et al., 2013) and at term, oxidative stress induced increased production of exosomes from fetal tissues may increase exosome quantity and thus signal strength. This study demonstrated that fetal signals can be carried as exosomal cargo through either diffusion between tissues or through systemic route from the fetal to the maternal side during pregnancy. This supports the postulate that fetal signals that can contribute to the initiation of human parturition can be delivered via exosomes.

## Author Contributions

RM and IB conceived and designed the experiments. SS-M and JL performed the experiments. RM, SS-M, and JL analyzed the data. RM, IB, GS, and CS contributed reagents/materials/analysis tools. RM, SS-M, JL, and IB wrote the paper.

## Conflict of Interest Statement

The authors declare that the research was conducted in the absence of any commercial or financial relationships that could be construed as a potential conflict of interest.
